# Predictive value of dynamic arterial elastance for vasopressor withdrawal: a systematic review and meta-analysis

**DOI:** 10.1186/s13613-024-01345-8

**Published:** 2024-07-09

**Authors:** Jorge Iván Alvarado-Sánchez, Sergio Salazar-Ruiz, Juan Daniel Caicedo-Ruiz, Juan José Diaztagle-Fernández, Yenny Rocio Cárdenas-Bolivar, Fredy Leonardo Carreño-Hernandez, Andrés Felipe Mora-Salamanca, Andrea Valentina Montañez-Nariño, María Valentina Stozitzky-Ríos, Carlos Santacruz-Herrera, Gustavo Adolfo Ospina-Tascón, Michael R. Pinsky

**Affiliations:** 1 Department of Intensive Care, Fundación Santa Fe de Bogotá, Bogotá, Colombia; 2 Department of Physiology Sciences, Faculty of Medicine, Universidad Nacional de Colombia, Bogotá, Colombia; 3 School of Medicine, Universidad del Rosario, Bogotá, Colombia; 4 Department of Internal Medicine, Fundación Universitaria de Ciencias de la Salud, Hospital de San José, Bogotá, Colombia; 5 Universidad de Los Andes, Bogotá, Colombia; 6 Department of Intensive Care, Fundación Valle del Lili, Cali, Colombia; 7 Translational Research Laboratory in Critical Care Medicine (TransLab-CCM), Universidad Icesi, Cali, Colombia; 8 Department of Critical Care Medicine, University of Pittsburgh, Pittsburgh, PA USA

**Keywords:** Critical care, Fluid therapy, Pulse pressure, Stroke volume, Blood flow velocity predictive value of tests, Systematic review

## Abstract

**Background:**

Dynamic arterial elastance (Ea_dyn_) has been investigated for its ability to predict hypotension during the weaning of vasopressors. Our study focused on assessing Ea_dyn_’s performance in the context of critically ill adult patients admitted to the intensive care unit, regardless of diagnosis.

**Main body:**

Our study was conducted in accordance with the Preferred Reported Items for Systematic Reviews and Meta-Analysis checklist. The protocol was registered in PROSPERO (CRD42023421462) on May 26, 2023. We included prospective observational studies from the MEDLINE and Embase databases through May 2023. Five studies involving 183 patients were included in the quantitative analysis. We extracted data related to patient clinical characteristics, and information about Ea_dyn_ measurement methods, results, and norepinephrine dose. Most patients (76%) were diagnosed with septic shock, while the remaining patients required norepinephrine for other reasons. The average pressure responsiveness rate was 36.20%. The synthesized results yielded an area under the curve of 0.85, with a sensitivity of 0.87 (95% CI 0.74–0.93), specificity of 0.76 (95% CI 0.68–0.83), and diagnostic odds ratio of 19.07 (95% CI 8.47–42.92). Subgroup analyses indicated no variations in the Ea_dyn_ based on norepinephrine dosage, the Ea_dyn_ measurement device, or the Ea_dyn_ diagnostic cutoff to predict cessation of vasopressor support.

**Conclusions:**

Ea_dyn_, evaluated through subgroup analyses, demonstrated good predictive ability for the discontinuation of vasopressor support in critically ill patients.

**Supplementary Information:**

The online version contains supplementary material available at 10.1186/s13613-024-01345-8.

## Introduction

In the intricate realm of circulatory shock management, striking a delicate equilibrium between sustaining mean arterial pressure (MAP) and enhancing cardiac output (CO) after initial fluid loading is pivotal. Traditionally, shock patients are administered vasopressor support and fluid therapy to maintain MAP. However, a nuanced challenge arises during the weaning of patients from vasopressor support, where a lack of clear predictive parameters for hypotension development complicates the clinical landscape. The dynamic arterial elastance (Ea_dyn_), derived from the ratio of pulse pressure variation (PPV) to stroke volume variation (SVV), could bridge this gap. Ea_dyn_ has emerged as a predictor of increased MAP after a fluid challenge in hypotensive volume-responsive patients [1, 2], suggesting that multifactorial insight is primarily associated with ventricular-arterial coupling [3–6]. A previous study delineated its link with left ventricular pulsatile load [3], positioning Ea_dyn_ as a predictor of vasopressor weaning without reactive hypotension. Notably, a randomized clinical trial assessing Ea_dyn_ clinical efficacy revealed a shortened vasopressor support duration and reduced acute kidney injury risk [7].

This study therefore aimed to evaluate the ability of the operative performance of Ea_dyn_ in critically ill adult patients to predict a subsequent reduction in MAP during the weaning of vasopressors. We also examined potential differences in the performance of the Ea_dyn_ according to the measurement methods and different clinical conditions.

## Methods

### Protocol

This systematic review and meta-analysis adhered to the Preferred Reporting Items for Systematic Reviews and Meta-Analysis (PRISMA) guidelines [8] and was registered in PROSPERO (registration number: CRD42023421462) in May 2023.

### Search strategy and data extraction

The MEDLINE and Embase databases were searched for all peer-reviewed articles published in May 2023 without publication date or language restrictions. Two independent researchers (J.I.A.S. and S.S.R.) reviewed potential studies according to the inclusion and exclusion criteria and extracted the data. Additionally, reference lists of selected manuscripts were manually scrutinized to identify potential studies that may not have been captured in the initial search. Keywords, index terms, and the detailed search strategy can be found in the protocol submitted to PROSPERO (registration number CRD42023421462, registered 26 May 2023).

### Study selection and inclusion criteria

Studies were selected according to the PICO framework as follows:

- P-Population: Critical care patients without any diagnostic restrictions.
- I – Index test: We included studies that evaluated the operative performance of Ea_dyn_ as a predictor of reactive hypotension during vasopressor weaning.
- C - Comparison: The analysis exclusively considered studies that included a well-defined criterion—specifically, a decrease in mean arterial pressure (MAP) following a reduction in norepinephrine dose—as the reference standard.
- O-Outcomes: We included studies that evaluated the operative performance of Ea_dyn_ as a predictor of vasopressor weaning support. When studies presented multiple datasets related to operative performance, all relevant information, including sensitivity, specificity, and area under the curve (AUC), was incorporated into the analysis.

### Exclusion criteria

Studies involving patients under 18 years of age, pregnant individuals, case reports, abstracts, and animal experiments were excluded.

### Study selection and data collection

Two authors (J.I.A.S. and S.S.R.) independently extracted the data in different spreadsheets; subsequently, the two spreadsheets were compared. Disagreements between the two authors were addressed through discussion. If a disagreement persisted, a third author reviewed the data extraction sheet to reach a consensus among all the authors.

### Data items

The data extracted from each clinical trial encompassed various parameters, including authors, year of publication, number of patients enrolled, type of patient, age, height, norepinephrine dose, diagnosis, APACHE II score, SOFA score, method used for Ea_dyn_ measurement, definition of positive responders, proportion of positive responders, diagnostic test cutoff point, pre- and post-norepinephrine weaning MAP values, mechanical ventilation requirement, tidal volume, lung compliance, positive end respiratory pressure (PEEP), airway driving pressure, diagnosis of acute respiratory distress syndrome (ARDS), presence of arrhythmias, specificity, sensitivity, and the Ea_dyn_ AUC.

### Risk of bias in individual studies

Two researchers (J.I.A.S. and S.S.R.) independently evaluated the risk of bias in the included studies using the QUADAS-2 tool [9]. Any disagreements between them were resolved through discussion with a third reviewer (J.J.D.F.). Additionally, the quality of evidence or the certainty of evidence was assessed using the GRADE framework [10].

### Statistical analysis

#### Analysis of individual studies

The sensitivity, specificity, and diagnostic odds ratio (DOR) were computed using a contingency table. The DOR provides a metric for assessing the discriminative ability of a diagnostic test, indicating how effectively it can distinguish between individuals with and without a specific condition. It is calculated as the ratio of the odds of true positives to false positives. A higher DOR suggests an increased probability that the test will yield true positive results compared to false positive results.

### Analysis of summary measures

Fitted sensitivity, specificity, and AUC data were evaluated through bivariate and hierarchical analyses. Receiver operating characteristic (ROC) curve summaries were calculated using the Rutter and Gatsonis method [11]. The AUC was graded according to Fisher et al. [12]. Heterogeneity among trials was gauged using Cochran’s Q tests, and its impact was quantified by calculating inconsistency (I2). An I2 (> 50%) indicates statistical significance [13]. A random effects model was used for the meta-analysis.

### Analysis of risk of bias across studies

Publication bias was assessed through a funnel plot. However, certain statistical tests were deemed inapplicable due to the limited number of included studies, rendering these tests impractical.

### Additional analysis

Some studies had several sets of operative performance data [7, 14]. In this situation, we also performed an analysis that included all the operative performance data. Subgroup analyses and random effect model meta-regression analyses were conducted based on various parameters: norepinephrine dose, diagnosis, APACHE II score, SOFA score, device used to measure SVV, device used to measure PPV, pressure responder definition, diagnostic test cutoff point, mechanical ventilation requirement, tidal volume, lung compliance, PEEP, airway driving pressure, diagnosis of acute respiratory distress syndrome (ARDS), and presence of arrhythmia. All operative performance data were included in the subgroup analyses.

Additionally, a sensitivity analysis was performed considering the risk of bias determined by QUADAS-2, the number of patients included in the studies, and the type of patient. The threshold effect was assessed using Spearman’s rank correlation coefficient and the Moses–Shapiro–Littenberg method [15]. R software, version 3.4.3, along with the mada and meta packages, was used for statistical analysis. The results are presented as 95% confidence intervals (CIs) and *p* values. A *p* value < 0.05 was considered to indicate statistical significance.

## Results

**Fig. 1:**
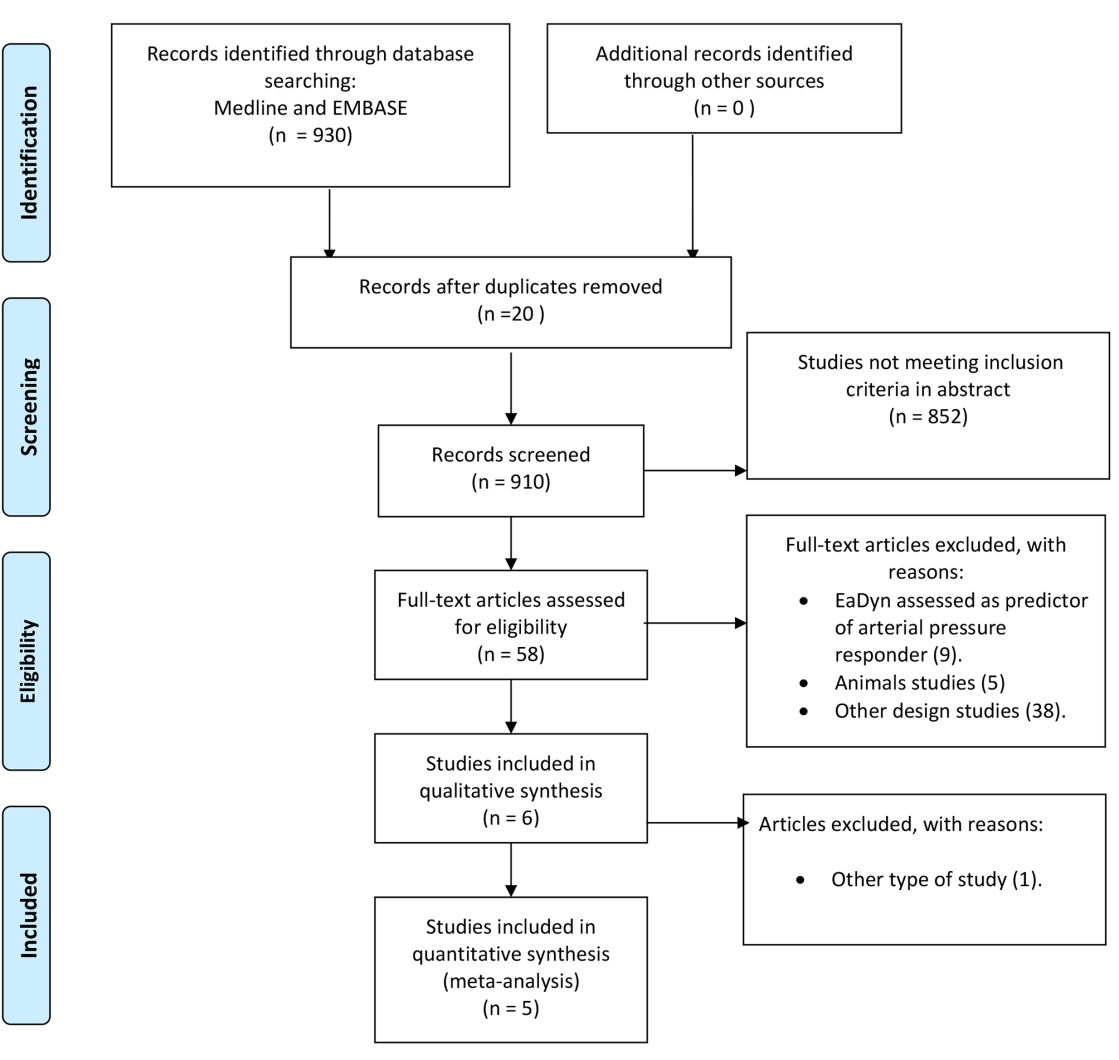
PRISMA Diagram

A total of 910 studies were gathered from the MEDLINE and Embase database searches. After applying the inclusion criteria, five studies met all the requirements and were included in the quantitative analysis [7, 14, 16–18] (Fig. 1).

Five of the clinical and Ea_dyn_ operative performance characteristics were evaluated, and the five studies involved a total of 183 patients (Tables 1 and 2). One hundred thirty-nine (139) patients were diagnosed with septic shock (76%), while the remaining forty-four patients required vasopressor support for other reasons (postoperative, *n* = 30 [16,4%]; polytrauma, *n* = 9 [4,9%]; hemorrhagic shock, *n* = 5 [2,7%]). A total of 183 vasopressor weaning procedures (one per patient) were cumulatively carried out, resulting in an average blood pressure responsiveness rate of 36.20%.

**Table 1:** General characteristics of selected studies

Study	year	Number of patients	Language	Type of patients	Device used to measure SVV	Device used to measure PPV	Responder definition	CUTOFF	Pressure responsiveness rate
Guinot et al.	2015	35	English	Sepsis	TPTD	TPTD	15%	0.94	0.37
Liang et al.	2017	32	Chinese	Sepsis	TPTD	TPTD	15%	0.97	0.40
Bar et al.	2018	35	English	Sepsis (40%), cardiovascular (40%), and others (20%).	NC-PCA	PCA	10%	0.9	0.31
Nguyen et al.	2021	39	English	Sepsis (41%), surgical (41%), and others (18%).	TTE	PCA	10%	0.8	0.3
Persona et al.	2023	42	English	Sepsis	NC-PCA	NC-PCA	10%	0.84	0.43

Values are expressed as pooled values (95% confidence interval). NC-PCA: non-calibrated pulse contour analysis; PPV: pulse pressure variation; PCA: pulse contour analysis; SVV: stroke volume variation; TPTD: transpulmonary thermodilution; TTE: transthoracic echocardiography

**Table 2:** Operative performance of dynamic arterial elastance as a predictor for the discontinuation of vasopressor support from included studies

Study	Year	True positive	Pressure responder	True negative	Non-pressure responder	Number of patients	Sensitivity	Specificity	AUC
Guinot et al.	2015	13	13	15	22	35	1	0.68	0.87
Liang et al.	2017	13	13	14	19	32	1	0.73	0.85
Bar et al.	2018	10	11	19	24	35	0.91	0.8	0.84
Nguyen et al.	2021	11	12	20	27	39	0.92	0.74	0.86
Persona et al.	2023	13	18	21	24	42	0.71	0.89	0.84

Values are expressed as pooled values or medians. AUC: area under curve reported by each study

### Risk of bias

**Fig. 2:**
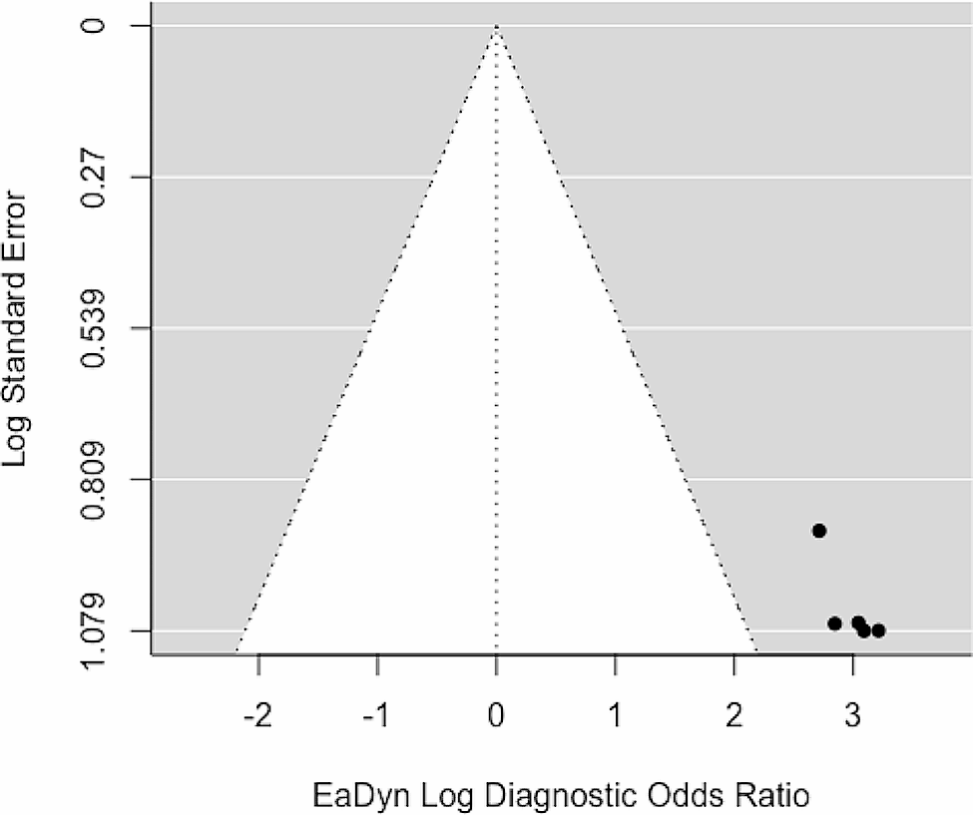
Funnel plot. Eadyn: dynamic arterial elastance

The five studies included in the study were classified as having a low risk of bias according to the QUADAS-2 tool (Additional file 1a). Funnel plot analysis revealed asymmetry in the included papers (Fig. 2). The GRADE assessment categorizes the certainty of the body of evidence as ‘moderate’ (Additional file 1b).

### Synthesis of results

**Fig. 3:**
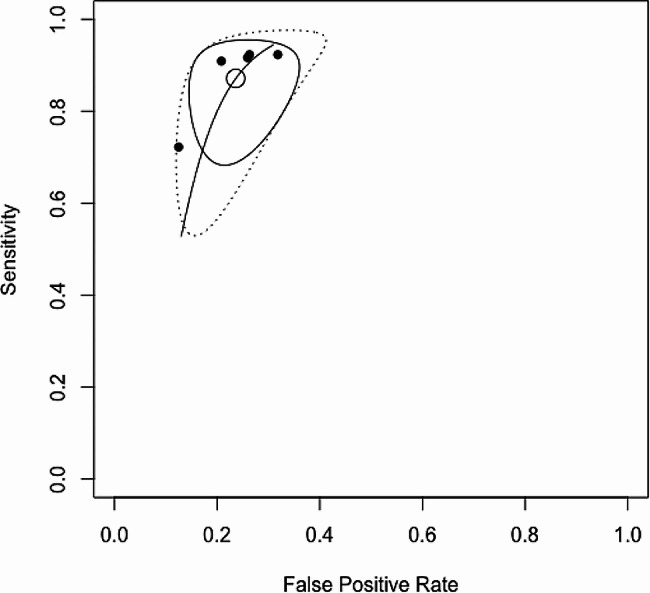
Receiver operating characteristic (ROC) curve summary for Dynamic arterial elastance

**Fig. 4:**
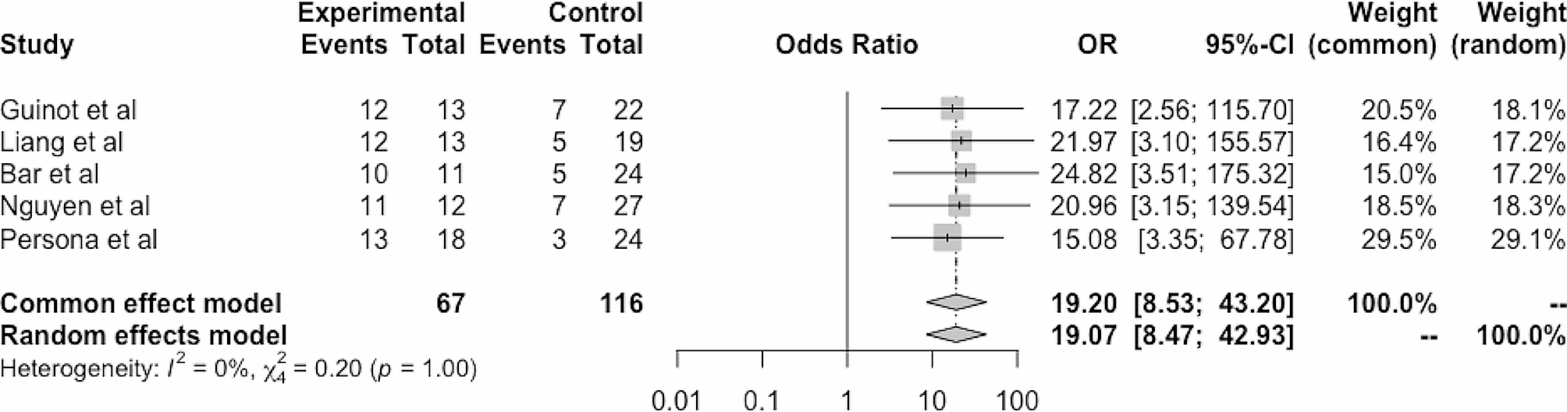
Forest plot. CI: Confidence interval; DOR: diagnostic odd ratio; I2: inconsistency

The estimated Ea_dyn_ operative performance was as follows: AUC = 0.85 (Fig. 3), sensitivity = 0.87 (95% CI = 0.74–0.93), specificity = 0.76 (95% CI = 0.68–0.83), and cutoff point = 0.89. The DOR was 19.07 (95% CI 8.47–42.92), and the I^2^ statistic for quantifying inconsistency among the included studies indicated that heterogeneity might not be important (I^2^ = 0%, Q = 0.20; *p* = 0.99) (Fig. 4).

### Additional analysis

We evaluated all the operative performance data reported by the included studies, encompassing a total of 9 sets of operative performance data. One study presented four sets of operative performance data [7], while another study included two sets [14] (Additional file 2). The Ea_dyn_ operative performance as a predictor of hypotension during the weaning of vasopressors was as follows: AUC, 0.85; sensitivity, 0.81 (95% CI, 0.64–0.91); specificity, 0.79 (95% CI, 0.73–0.84); and cutoff point, 0.85. The DOR was 14.46 (95% CI 8.00-26.15), and the I^2^ statistic suggested that heterogeneity might not be important (I2 = 0%, Q = 3.14; *p* = 0.92).

According to the evaluation of the 9 operative performance datasets, the operative performance of Ea_dyn_ was unaffected by variations in the norepinephrine dose (by meta-regression), the PPV measuring device, the SVV measuring device, the pressure responder definition, or the diagnostic cutoff point (*p* > 0.05) (Table 3). Subgroup analysis for the remaining variables was omitted due to missing or insufficient data in certain studies. Furthermore, sensitivity analysis based on the number of patients indicated no changes in the operative performance of Eadyn (DOR = 1.12 + 1.03 per number of patients, *p* > 0.57). A sensitivity analysis based on the QUADAS-2 was not conducted because all the included studies exhibited a low risk of bias. Spearman’s rank correlation test indicated the presence of a threshold effect (*r* = 0.80, *p* < 0.05). However, upon conducting a meta-regression between DOR and the cutoff values of each study, no such effect was discerned (Table 3).

**Table 3:** Subgroup analysis

Subgroup	Number datasets	Crude DOR (95%CI)	Adjusted DOR (95% CI)	*P*-value by subgroup analysis	*P*-value by meta-regression	I^2^(%)
EaDyn cut-off point	9	18.82(9.73–36.41)	DOR = 7.93 + 0.42 (cutoff%)	NA	0.68	0
SVV measuring device. TPTD NC-PCA TTE	6 2 1	18.82(9.73–36.41)	16.20 (7.17–36.60) 23.16(6.29–85.31) 31.43(3.41-289.58)	0.81	NA	0
PPV measuring device. TPTD Analysis of arterial tracing	5 4	18.82(9.73–36.41)	13.77(5.75–32.97) 28.53(10.4–78.1)	0.28	NA	0
Pressure responder definition. MAP < 15%. MAP < 10%.	5 4	18.82(9.73–36.41)	13.77(5.75–32.97) 28.52(10.42–78.10)	0,28	NA	0
Noradrenaline dose	9	17.85(8.96–35.57)	DOR = 8.95 + 0.02 (mcg/Kg/min)	NA	0.27	0

Values are expressed as pooled data. CI: confidence interval; DOR: diagnostic odd ratio; EaDyn, dynamic arterial elastance; I^2^: inconsistency; MAP: mean arterial pressure; NC-PCA: non-calibrated pulse contour analysis; PEEP, positive end-expiratory pressure; PPV, pulse pressure variation; SVV, stroke volume variations; TPTD: transpulmonary thermodilution; TTE: transthoracic echocardiography

## Discussion

Our study revealed that Ea_dyn_ serves as a good predictor of MAP reduction during vasopressor weaning. Considering our findings, an Eadyn value greater than 0.89 predicts no reduction in mean arterial pressure during the weaning of vasopressor support. Additionally, we observed that different SVV measurement methods employed for Ea_dyn_ estimation consistently demonstrated comparable operative performance.

The assessment of arterial load is intricate and involves factors such as pulsatile and steady components, which, in turn, depend on other hemodynamic variables. For instance, systemic vascular resistance and the MAP are associated with the steady component, while arterial variables (impedance, elastance, and compliance) and wave reflection are linked with the pulsatile component [3–6, 19]. The resultant MAP is contingent upon the interplay between cardiac and arterial elements. Under typical pressure conditions and when preload dependence is present, the SVV aligns with the PPV, causing Ea_dyn_ to approach 1. Conversely, in clinical scenarios characterized by low arterial load and preload dependence, Ea_dyn_ is < 1, while with increased vasomotor tone, Ea_dyn_ is often > 1.5 [3, 4, 6]. This interaction is particularly dependent on arterial compliance because the PPV is primarily altered by arterial compliance [3]. Notably, the operative performance of Ea_dyn_ serves as a predictor of an increase in MAP after a fluid challenge in hypotensive critically ill patients, where arterial compliance is fixed and decreases with the use of norepinephrine [1, 20–22]. However, the operative performance of Ea_dyn_ is poorer in surgical patients in whom norepinephrine is not frequently used and in whom arterial compliance could be high or normal [23–26]. Additionally, the relationship between Ea_dyn_ and arterial compliance has allowed us to assess the use of the Ea_dyn_ as a predictor of MAP during the weaning of critically ill adult patients [7, 14, 16, 18].

It is important to emphasize that Ea_dyn_ is correlated with vascular waterfall (WV, a pressure essential for maintaining tissue perfusion during periods of low blood flow) and critical closing pressure (CCP, the arterial pressure at which blood flow is halted owing to arteriole occlusion) [27]. The augmentation of VW and CCP, facilitated by the administration of norepinephrine, contributes to an enhancement in tissue perfusion [28]. In alignment with the aforementioned findings, the Ea_dyn_ can serve as a variable that elucidates the intricate relationship between cardiac function and arterial load, delineates the effects of hemodynamic treatment on arterial load, and reveals hemodynamic coherence. This assertion was supported by the findings of a clinical trial in which the use of Ea_dyn_ as a hemodynamic tool for vasopressor weaning demonstrated a reduction of duration of vasopressor support, the length of hospital stay, and the incidence of renal failure [29, 30].

Our meta-analysis revealed several interesting findings and raised new research questions. First, a consistent and favorable predictive performance was observed in critically ill patients, and these findings are homogeneous, suggesting that the findings can be extrapolated to general clinical settings. Second, the device used to measure the SVV and calculate the Ea_dyn_ did not affect the operative performance. We included studies that used calibrated pulse analysis contours [7], transthoracic echocardiography [14], and uncalibrated pulse analysis contours [16, 18]. This is important because the SVV is usually derived from the arterial pressure waveform, so inherent covariance of PPV and SVV changes can occur. Thus, the consistency of Ea_dyn_ across measurement methods attests to the robustness of the parameter. Finally, no studies have evaluated the operative efficacy of Ea_dyn_ in patients treated with vasopressor drugs other than norepinephrine. However, we would expect that their responses would be similar.

Our study has certain limitations. First, the inclusion of a limited number of studies raises the possibility of publication bias and heterogeneity among the included studies, underscoring the need for additional research. Second, certain clinical scenarios, such as hypovolemic and neurogenic shock, were not assessed. Consequently, the generalizability of our findings to these specific conditions is limited, highlighting the importance of further research addressing the usefulness of Ea_dyn_ in such clinical contexts.

## Conclusions

Our study concludes that the Ea_dyn_ operative performance is good to predict hypotension during the weaning of vasopressors in critically ill adult patients, particularly in septic shock patients. The consistency of the results, given the high methodological quality of the included studies, supports our findings. Despite the need for further evaluation of the Ea_dyn_ evaluation in other clinical scenarios, our results suggest that the Ea_dyn_ has potential as a predictive tool for optimizing vasopressor weaning strategies. These findings may provide useful insights for improving clinical decision-making and patient outcomes in critical care settings.

### Electronic supplementary material

Below is the link to the electronic supplementary material.

Supplementary Material 1

Supplementary Material 2

Supplementary Material 3

## Data Availability

All the data generated and analyzed during this study are available from the corresponding author upon reasonable request. The studies included in this systematic review are available from their corresponding author and journal.
