# Supplementary material for: Predictive value of dynamic arterial elastance for vasopressor withdrawal: a systematic review and meta-analysis
Source: Ann Intensive Care. 2024 Jul 9;14:108. doi: 10.1186/s13613-024-01345-8 (PMC11233481; doi:10.1186/s13613-024-01345-8)
Supplement: Supplementary file 1 — Supplementary Material 1 [file 13613_2024_1345_MOESM1_ESM.docx]

# **Predictive Value of Dynamic Arterial Elastance for Vasopressor Withdrawal: A Systematic Review and Meta-analysis.**

Additional file 1

Jorge Iván Alvarado-Sánchez^1, 2^ (ORCID: 0000-0003-4320-3150)

Sergio Salazar-Ruiz^1-3^ (ORCID: 0009-0003-1006-725X)

Juan Daniel Caicedo-Ruiz^2^

Juan José Diaztagle-Fernández^2,4^

Yenny Rocio Cárdenas-Bolivar^1^

Fredy Leonardo Carreño-Hernandez^5^

Andrés Felipe Mora-Salamanca^1^ (ORCID: 0000-0003-1509-0080)

Andrea Valentina Montañez-Nariño^1^

Maria Valentina Stozitzky-Ríos^1^

Carlos Santacruz-Herrera^1^

Gustavo Adolfo Ospina-Tascón ^6,7^ (ORCID: 0000-0001-9370-3298)

Michael R Pinsky ^8^

1. Department of Intensive Care, Fundación Santa Fe de Bogotá, Bogotá, Colombia.
2. Department of Physiology Sciences, Faculty of Medicine, Universidad Nacional de Colombia, Bogotá, Colombia.
3. School of Medicine, Universidad del Rosario, Bogotá, Colombia
4. Fundación Universitaria de Ciencias de la Salud, Bogotá, Colombia. Department of Internal Medicine, Hospital de San José, Bogotá, Colombia.
5. Universidad de Los Andes, Bogotá, Colombia.

6. Department of Intensive Care, Fundación Valle del Lili, Cali, Colombia.

7. Translational Research Laboratory in Critical Care Medicine (TransLab-CCM), Universidad Icesi, Cali, Colombia

8. Department of Critical Care Medicine, University of Pittsburgh, Pittsburgh, PA, USA

Corresponding author: Jorge Iván Alvarado Sánchez: [Jialvarados@unal.edu.co](mailto:Jialvarados@unal.edu.co)

**Additional file 1a. Risk of bias of the trials as assessed by QUADAS-2 criteria.**

| Study | Year | Risk of bias/patient selection | Risk of bias/index test | Risk of bias/Reference Standard | Risk of bias/Flow and timing | Applicability/patient selection | Applicability/index test | Applicability/Reference Standard |
| --- | --- | --- | --- | --- | --- | --- | --- | --- |
| Guinot et al | 2015 | Low | Low | Low | Low | Low | Low | Low |
| Liang et al | 2017 | Low | Low | Low | Low | Low | Low | Low |
| Bar et al | 2018 | Low | Low | Low | Low | Low | Low | Low |
| Nguyen et al | 2021 | Low | Low | Low | Low | Low | Low | Low |
| Persona et al | 2023 | Low | Low | Low | Low | Low | Low | Low |

**Additional file 1b. GRADE assessment of included studies.**

**Question**: Should dynamic arterial elastance be used to screen for weaning from vasopressor support in critically ill adult patients??

| \| Sensitivity \| 0.87 (95% CI 0.74-0.93) \| \| --- \| --- \| \| Specificity \| 0.76 (95% CI: 0.68-0.83) \| |  | \| Prevalences \| 36.20% \|  \|  \| \| --- \| --- \| --- \| --- \| |  |
| --- | --- | --- | --- | --- | --- | --- | --- | --- | --- | --- | --- |

| Outcome | № of studies (№ of patients) | Study design | Factors that may decrease certainty of evidence | | | | | Effect per 100 patients tested | Test accuracy CoE |
| --- | --- | --- | --- | --- | --- | --- | --- | --- | --- |
|  |  |  | Risk of bias | Indirectness | Inconsistency | Imprecision | Publication bias | pre-test probability of35.52% |  |
| **True positives** (patients with weaning from vasopressor support) | 5 studies 67 patients | cross-sectional (cohort type accuracy study) | not serious | not serious | not serious | not serious | publication bias strongly suspected | 30 (26 to 33) | ⨁⨁⨁◯ Moderate |
| **False negatives** (patients incorrectly classified as not having weaning from vasopressor support) |  |  |  |  |  |  |  | 6 (3 to 10) |  |
| **True negatives** (patients without weaning from vasopressor support) | 5 studies 116 patients | cross-sectional (cohort type accuracy study) | not serious | not serious | not serious | not serious | publication bias strongly suspected | 49 (44 to 54) | ⨁⨁⨁◯ Moderate |
| **False positives** (patients incorrectly classified as having weaning from vasopressor support) |  |  |  |  |  |  |  | 15 (10 to 20) |  |

CI: Confidence interval
