# Supplementary material for: Predictive value of dynamic arterial elastance for vasopressor withdrawal: a systematic review and meta-analysis
Source: Ann Intensive Care. 2024 Jul 9;14:108. doi: 10.1186/s13613-024-01345-8 (PMC11233481; doi:10.1186/s13613-024-01345-8)
Supplement: Supplementary file 2 — Supplementary Material 2 [file 13613_2024_1345_MOESM2_ESM.docx]

# **Predictive Value of Dynamic Arterial Elastance for Vasopressor Withdrawal: A Systematic Review and Meta-analysis.**

Additional file 2

Jorge Iván Alvarado-Sánchez^1, 2^ (ORCID: 0000-0003-4320-3150)

Sergio Salazar-Ruiz^1-3^ (ORCID: 0009-0003-1006-725X)

Juan Daniel Caicedo-Ruiz^2^

Juan José Diaztagle-Fernández^2,4^

Yenny Rocio Cárdenas-Bolivar^1^

Fredy Leonardo Carreño-Hernandez^5^

Andrés Felipe Mora-Salamanca^1^ (ORCID: 0000-0003-1509-0080)

Andrea Valentina Montañez-Nariño^1^

Maria Valentina Stozitzky-Ríos^1^

Carlos Santacruz-Herrera^1^

Gustavo Adolfo Ospina-Tascón ^6,7^ (ORCID: 0000-0001-9370-3298)

Michael R Pinsky ^8^

1. Department of Intensive Care, Fundación Santa Fe de Bogotá, Bogotá, Colombia.
2. Department of Physiology Sciences, Faculty of Medicine, Universidad Nacional de Colombia, Bogotá, Colombia.
3. School of Medicine, Universidad del Rosario, Bogotá, Colombia
4. Fundación Universitaria de Ciencias de la Salud, Bogotá, Colombia. Department of Internal Medicine, Hospital de San José, Bogotá, Colombia.
5. Universidad de Los Andes, Bogotá, Colombia.

6. Department of Intensive Care, Fundación Valle del Lili, Cali, Colombia.

7. Translational Research Laboratory in Critical Care Medicine (TransLab-CCM), Universidad Icesi, Cali, Colombia

8. Department of Critical Care Medicine, University of Pittsburgh, Pittsburgh, PA, USA

Corresponding author: Jorge Iván Alvarado Sánchez: [Jialvarados@unal.edu.co](mailto:Jialvarados@unal.edu.co)

**Additional file 2. General characteristics of all operative performance data of selected studies**

| **Study** | **year** | **Number of patients** | **Type of patients** | **Device used to measure SVV** | **Device used to measure PPV** | **Responder definition** | **CUTOFF** | **Sensitivity** | **Specificity** | **AUC** | **Pressure responsiveness rate** |
| --- | --- | --- | --- | --- | --- | --- | --- | --- | --- | --- | --- |
| Guinot et al | 2015 | 35 | Sepsis | TPTD | TPTD | 15% | 0.94 | 1 | 0.68 | 0.87 | 0.37 |
| Guinot et al | 2015 | 35 | Sepsis | TPTD | TPTD | 15% | 0.83 | 0.77 | 0.73 | NR | 0.37 |
| Guinot et al | 2015 | 35 | Sepsis | TPTD | TPTD | 15% | 0.77 | 0.69 | 0.86 | NR | 0.37 |
| Guinot et al | 2015 | 35 | Sepsis | TPTD | TPTD | 15% | 0.7 | 0.23 | 1 | NR | 0.37 |
| Liang et al | 2017 | 32 | Sepsis | TPTD | TPTD | 15% | 0.97 | 1 | 0.73 | 0.85 | 0.40 |
| Bar et al | 2018 | 35 | Sepsis and cardiovascular | NC-PCA | PCA | 10% | 0.9 | 0.91 | 0.8 | 0.84 | 0.31 |
| Nguyen et al | 2021 | 39 | Sepsis and surgical | TTE | PCA | 10% | 0.8 | 0.92 | 0.74 | 0.8 | 0.3 |
| Nguyen et al | 2021 | 39 | Sepsis and surgical | TPTD | PCA | 10% | 0.9 | 0.93 | 0.79 | 0.86 | 0.3 |
| Persona et al | 2023 | 42 | Sepsis | NC-PCA | NC-PCA | 10% | 0.84 | 0.71 | 0.89 | 0.84 | 0.43 |

Values are expressed as pooled values (95% confidence interval). NC-PCA, non-calibrated pulse contour analysis; PPV, pulse pressure variation; PCA, pulse contour analysis; SVV, stroke volume variation; TPTD, transpulmonary thermodilution; TTE, transthoracic echocardiography; AUC, area under curve reported by each study; NE, not reported.
