# Supplementary material for: Predictive value of dynamic arterial elastance for vasopressor withdrawal: a systematic review and meta-analysis
Source: Ann Intensive Care. 2024 Jul 9;14:108. doi: 10.1186/s13613-024-01345-8 (PMC11233481; doi:10.1186/s13613-024-01345-8)
Supplement: Supplementary file 3 — Supplementary Material 3 [file 13613_2024_1345_MOESM3_ESM.docx]

Caption for Figures:

**Figure 1. PRISMA Diagram.** Ea_dyn_: dynamic arterial elastance.

**Figure 2. Funnel plot.** Ea_dyn_: dynamic arterial elastance.

**Figure 3. Receiver operating characteristic (ROC) curve summary for Dynamic arterial elastance.**

**Figure 4. Forest plot.** CI: Confidence interval; DOR: diagnostic odd ratio; I^2^: inconsistency.
